# Nanoporous electroporation needle for localized intracellular delivery in deep tissues

**DOI:** 10.1002/btm2.10418

**Published:** 2022-12-08

**Authors:** Gyeong Won Lee, Byeongyeon Kim, Tae Wook Lee, Sang‐Gu Yim, Ajeesh Chandrasekharan, Hyewon Kim, Sungyoung Choi, Seung Yun Yang

**Affiliations:** ^1^ Department of Biomaterials Science (BK21 Four Program) Pusan National University Miryang South Korea; ^2^ Department of Biomedical Engineering, Department of Electronic Engineering, Hanyang Institute of Bioscience and Biotechnology Hanyang University Seoul South Korea

**Keywords:** electric pulse‐driven drug delivery, electroporation, intracellular delivery, nanopore membrane

## Abstract

The exogenous control of intracellular drug delivery has been shown to improve the overall efficacy of therapies by reducing nonspecific off‐target toxicity. However, achieving a precise on‐demand dosage of a drug in deep tissues with minimal damage is still a challenge. In this study, we report an electric‐pulse‐driven nanopore‐electroporation (nEP) system for the localized intracellular delivery of a model agent in deep tissues. Compared with conventional bulk electroporation, in vitro nEP achieved better transfection efficiency (>60%) with a high cell recovery rate (>95%) under a nontoxic low electroporation condition (40 V). Furthermore, in vivo nEP using a nanopore needle electrode with a side drug‐releasing compartment offered better control over the dosage release, time, and location of propidium iodide, which was used as a model agent for intracellular delivery. In a pilot study using experimental animals, the nEP system exhibited two times higher transfection efficiency of propidium iodide in the thigh muscle tissue, while minimizing tissue damage (<20%) compared to that of bulk electroporation. This tissue‐penetrating nEP platform can provide localized, safe, and effective intracellular delivery of diverse therapeutics into deep tissues in a controlled manner.

## 1 INTRODUCTION

Conventional oral and intravenous routes of drug administration are the most common approaches for achieving systemic and local drug delivery for the treatment of various diseases.^1^, ^2^, ^3^, ^4^ Most of the drugs introduced into the body pass through the liver during metabolism and are then released from the body, causing drug‐induced liver injury during this process.^5^, ^6^ Some cell‐impermeable drugs require additional processing for increasing the intracellular delivery to the cell membrane. Thus, noninvasive localized drug delivery with enhanced cell permeation can improve therapeutic outcomes for diseases predominantly triggered by the disorder and malfunction of cells.^7^, ^8^ In addition, introducing drugs and biomolecules into cells is an important strategy to understand cellular mechanisms, as it allows to decipher cellular functions, direct cell fate, and reprogram cell behaviors.^7^

Several methods have been developed to breach the cell membrane for drug delivery into cells, including viral vectors and chemical methods.^9^, ^10^, ^11^, ^12^, ^13^ While viral vectors are effective in delivering biomolecules into cells, they suffer from certain limitations, mainly immunogenicity.^14^ In contrast, chemical methods (involving formation of nanosized complexes with lipids or polymers) are often limited by toxicity and quality control.^8^ In addition, the delivery method for injecting chemical complexes can suffer from low delivery efficiency owing to endosomal entrapment that occurs during endocytosis.^15^, ^16^, ^17^ Alternatively, physical approaches, including microinjection, biolistics, jet injection, ultrasound, and electroporation, have also been utilized to transiently modify cell membrane permeability and have gained considerable attention owing to their technical simplicity and operational flexibility.^18^, ^19^ Among these, electroporation (EP) has received significant attention because of its high efficacy in terms of the intracellular delivery of exogenous therapeutic agents for a broad range of cell types as well as its facile integration into medical devices and equipment.^20^ The application of an electric field gradient (≈1 kV/cm) to cells typically generates transient membrane pores and increases the permeability of the cell membrane, thus allowing direct intracellular delivery of cell‐impermeable materials.^21^, ^22^, ^23^ Due to its rapid and immediate delivery characteristics, conventional bulk EP has been widely used in biomedical applications ranging from cellular dedifferentiation to electrochemotherapy.^24^, ^25^, ^26^, ^27^ However, bulk EP using plate or needle electrodes suffers from significant cell damage and random, low transfection efficiency, which may be attributed to the nonuniform generation of high‐voltage pulses between the electrodes.^28^, ^29^

Alternatively, nanopore‐electroporation (nEP) has been developed to achieve efficient intracellular drug delivery under low‐voltage conditions with improved cell viability.^30^, ^31^, ^32^, ^33^, ^34^, ^35^, ^36^ By inducing a focused electric field through the nanopore, perforation can occur only in a small fraction of the cell membrane, thereby improving cell viability. In addition, since charged molecules can be directly injected into cells by nEP, the intracellular delivery efficiency of nEP is more than 10‐fold higher than that of bulk EP systems.^31^ Upon integration into microchannel devices, the nEP system could be used for single‐cell transfection with high efficiency and precise delivery.^37^ Compared with bulk EP, nEP generates localized and well‐defined pores on the cell membrane to deliver charged gene‐editing agents, such as plasmids, thus yielding high cell viability and transfection efficiency.^29^ Notably, tissue nanotransfection chips fabricated by multiple semiconductor lithography techniques demonstrated promising results for in vivo tissue reprogramming following pulsed nEP.^30^, ^38^, ^39^ However, the in vivo application of nEP systems with 2D planar designs is limited to the body surface (mainly the skin surface), as nEP is effective on cells that are in direct contact with the nanopores. Furthermore, for transdermal cargo delivery by nEP, pretreatments, such as microneedling^40^ or exfoliation procedure^30^, ^38^, ^39^ are required for having a closer interface between the planar nEP electrode and deeper layers of the skin.

A suitable platform to overcome the limitations of existing nEP‐based intracellular delivery of functional molecules should (i) effectively permeabilize the cell membrane under a nontoxic working voltage, (ii) be applicable for deep tissues without the pretreatment of the target tissues, (iii) offer reliable and pulse‐responsive intracellular delivery with minimal tissue damage, (iv) be a simple and scalable format for application in a large area, and (v) be able to integrate with the current EP system for easy translation.

**FIGURE 1:**
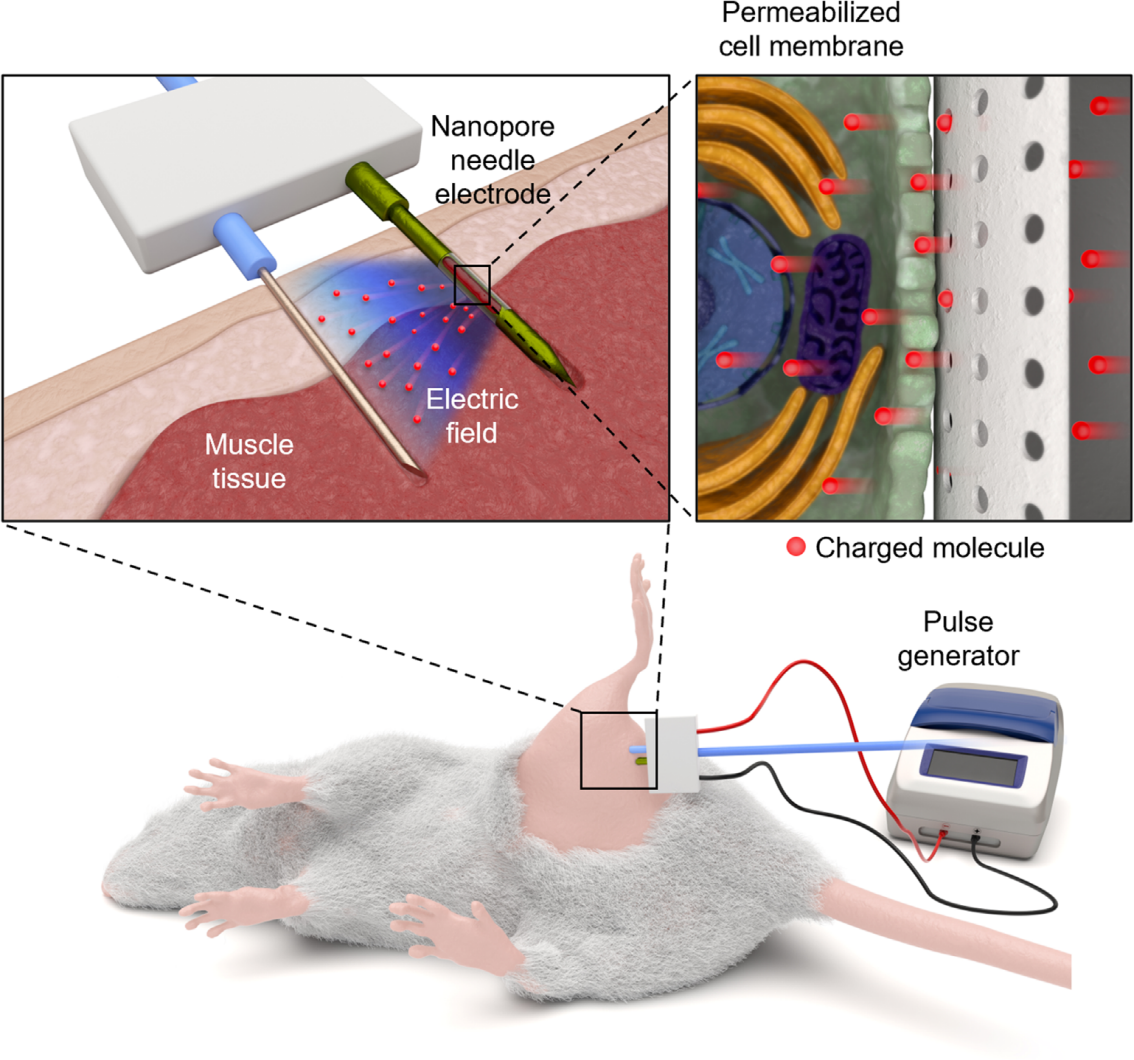
Schematic illustration of the in vivo nEP system. That consisting of a conventional metal needle and nanopore needle electrode with a side drug‐releasing compartment, applied into the thigh muscle of a mouse. Application of an electric field through the nanopores of the needle electrode generates transient pores on the cell membrane. The charged molecules accelerated through electrical stimulation are released through the nanopores, thereby enabling intracellular delivery of charged target molecules through the temporarily perforated cell membrane.

Given this background, in this study, we attempted to design an in vivo nEP platform based on a nanopore needle electrode fabricated by the merging of micro‐ and nano‐manufacturing technologies for low tissue damage and high transfection efficiency (Figure 1). This in vivo nEP system controlled the release amount, release time, and release location of the target molecules in a better way for intracellular delivery upon electrical stimulation. We first developed an in vitro testing device for nEP to identify the optimal electric‐field conditions for effective intracellular delivery. We then engineered the nEP system with 2‐needle array electrodes consisting of a metal needle and a nanopore needle electrode with a side drug‐releasing compartment for in vivo deep tissue application. This nanopore electrode can effectively focus the electric field, which can only be applied to cells around the nanopores. The constructed nEP system was investigated to assess the efficacy of intracellular delivery to deep tissues. The safety and efficiency of this nEP system were verified by applying it to the thigh muscle of a mouse, and then examining the extent of tissue damage as well as the transfected area with a cell‐impermeable dye as a model agent.

## 2 RESULTS AND DISCUSSION

### 2.1 In vitro testing device for nEP

**FIGURE 2:**
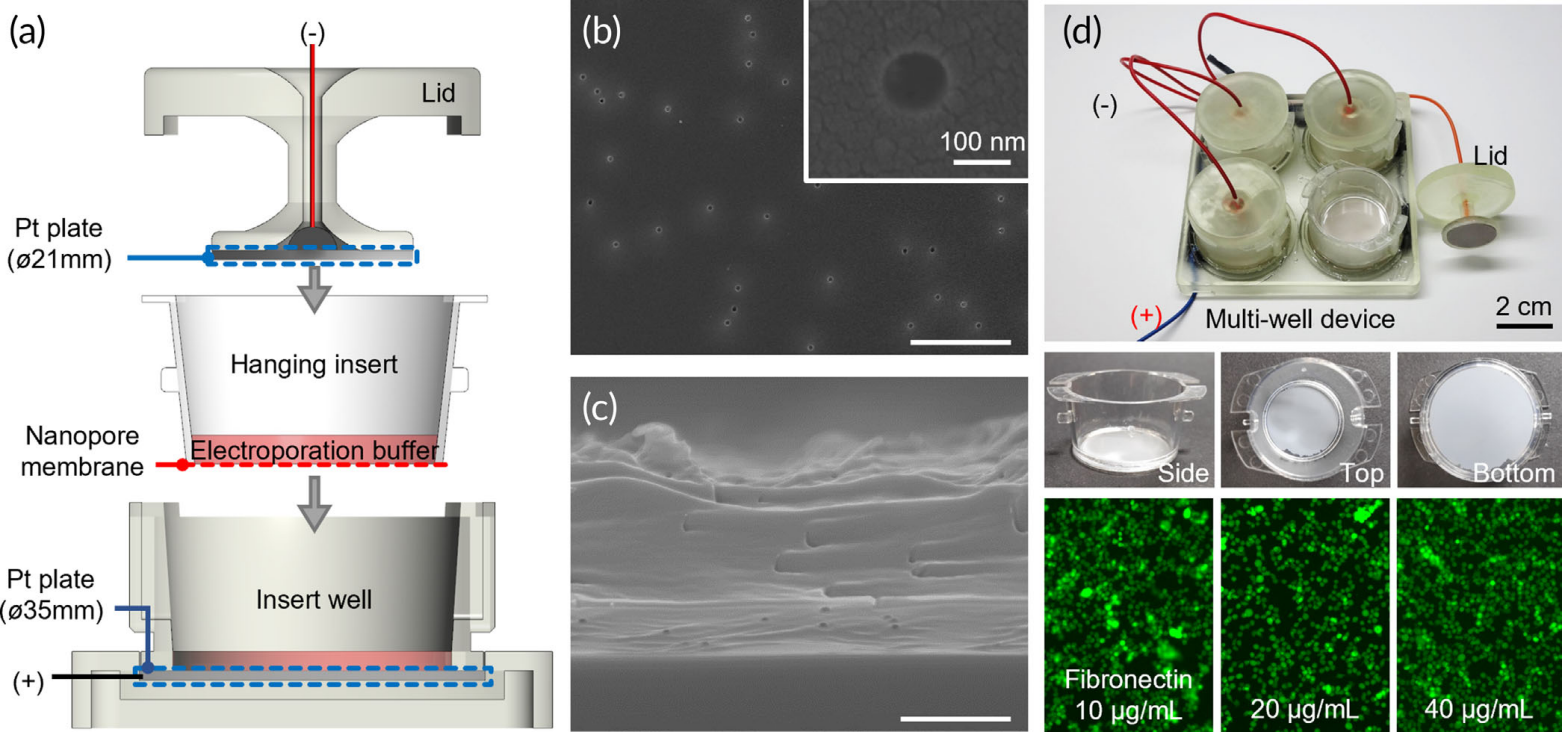
In vitro testing device for nEP. (a) Schematic showing the device structured by sandwiching cells cultured on a nanopore membrane between two Pt plate electrodes. (b) Top‐surface and (c) cross‐sectional SEM images of the nanopore membrane with cylindrical uniform pore structure (scale bar: 5 μm). (d) Fabricated in vitro testing device and cells cultured on the membrane. (Top) Photograph of the multi‐well nEP device. (Middle) Photographs of the hanging insert with the attached nanopore membrane. (Bottom) Fluorescence micrographs showing differences in the adhesion of cells grown on the nanopore membrane depending on the concentration of the cell adhesion molecule, fibronectin. Cells were fluorescently visualized using Calcein AM staining.

We first engineered an in vitro testing device to verify the principle of nEP and screen the electrical stimulation conditions before exploring the usage of nEP in in vivo experiments (Figure 2). In principle, nEP using DC voltage pulses was selectively applied through a nanopore membrane only to cells in contact with the nanopores. Thus, (1) the area in which the cells are exposed to the electric field can be limited to the size of the nanopore, and (2) most of the DC voltage is applied only to the nanoporous membrane, which has a relatively higher resistance than the EP medium. In the case of conventional bulk EP, there is a clear trade‐off between the EP efficiency and cell survival rate; when the voltage is increased to improve the efficiency, the cell survival rate markedly decreases. In contrast, nEP can decouple this relationship by adjusting the density and pore diameter of the nanopores and controlling the area of the electric field applied to single cells. In other words, even if a strong electric field is applied, cell damage can be minimized by reducing the area in which the field is applied to a single cell. In addition, electrolysis, a chronic issue of conventional bulk EP, can be considerably suppressed by increasing the electrical resistance of the overall EP system with the addition of the nanopore membrane, thereby decreasing the corresponding electric current. Although the applied voltage can be largely focused on the nanopore membrane, the resistance of the relatively bulky EP medium cannot be neglected. Therefore, to apply uniform electric strength to the nanopores distributed in the nanopore membrane with a diameter of 25 mm, the nanopore membrane was placed between two circular plate electrodes with diameters of 21 mm (top) and 35 mm (bottom) (Figure 2a). The nanopore membrane prepared by track‐etching of the polycarbonate film had an average pore diameter of 100 nm with a narrow pore size distribution (Figure S1) and a pore density of 1 × 10^7^ pores/cm^2^ (Figure 2b). The cylindrical nanopores spanned the entire membrane thickness of 7 μm (Figure 2c). The device was fabricated in a multi‐well format to improve screening throughput; the hydrophobic surface of the nanopore membrane was coated with a cell adhesion molecule, fibronectin, at a concentration of 20 μg/mL or higher to improve cell adhesion (Figure 2d).

### 2.2 Simulation study of bulk EP and nEP

**FIGURE 3:**
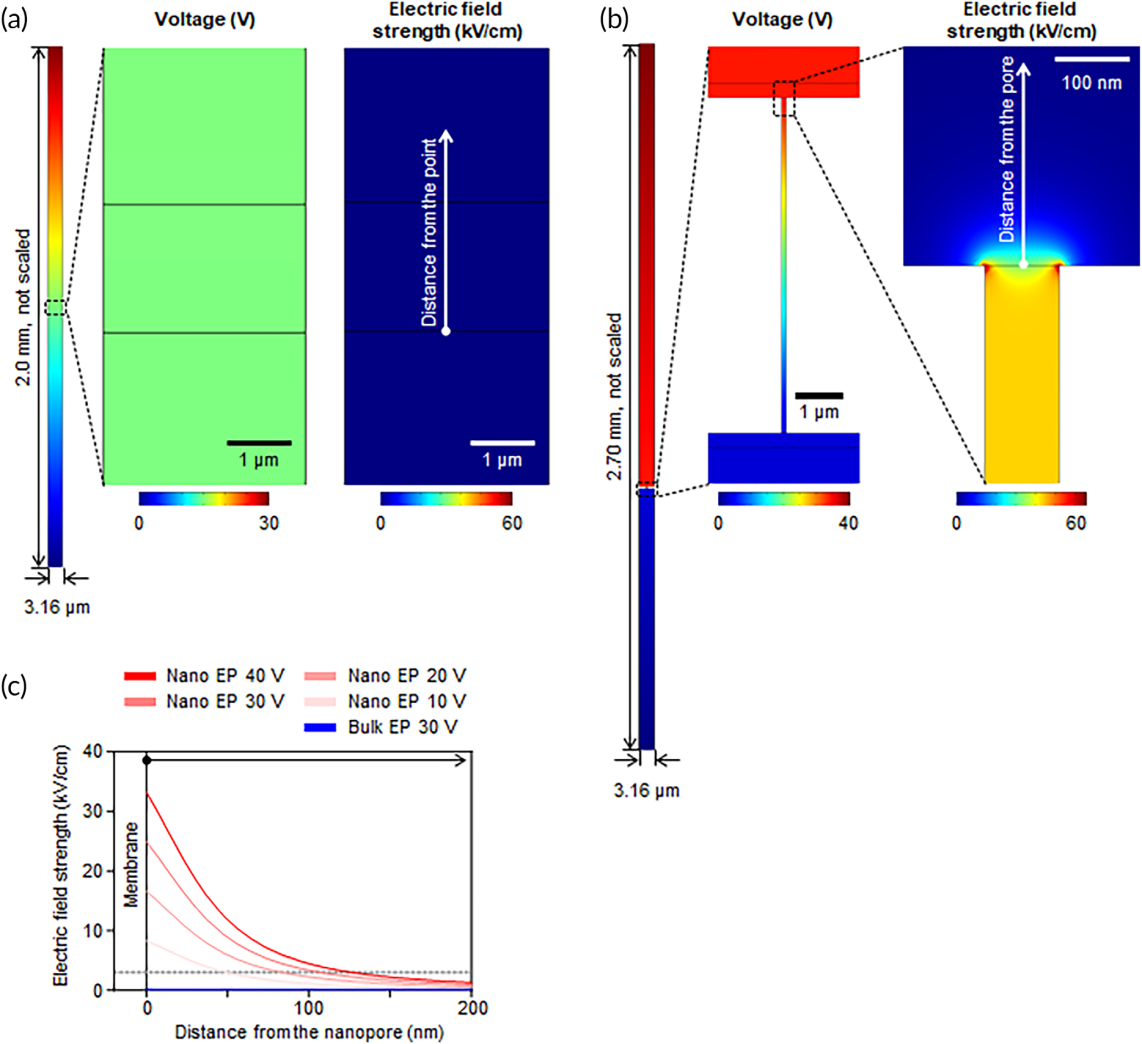
Simulation results showing the electric field distributions in the bulk EP and nEP. Since the nanopores on the membrane can be considered as parallel resistors in an electric circuit, a single nanopore was used for electric field simulation. (a) The parallel plate electrodes in a homogeneous fluid generate a uniform electric field, while (b) the addition of the nanopore membrane results in electric field getting focused to the membrane. The applied voltages were 30 V and 40 V for bulk EP and nEP, respectively. (c) Electric field strength profiles along a direction away from a certain point in the bulk EP and nanopore entrance in the nEP.

We performed simulation studies to compare the electric field distributions in bulk EP and nEP devices. The bulk EP device is an electroporation cuvette composed of two parallel plate electrodes spaced 2 mm apart, whereas the nEP device consists of two circular electrodes spaced 2.7 mm apart. Since the magnitude of the electric field is inversely proportional to the distance between the electrodes, a smaller inter‐electrode gap produces a higher electric field under the same voltage applied. To match the voltage per unit length between the two electrodes of each device, it was necessary to multiply the bulk EP voltage by 1.35 times. For example, 30 V in the bulk EP corresponds to 40.5 V in the nEP. As simulated by COMSOL Multiphysics, the electric field in the bulk EP was uniform between the plate electrodes, whereas the electric field in the nEP was locally focused on a thin membrane with a thickness of 7 μm (Figure 3a,b). This is due to the local increase in electrical resistance upon the addition of the nanopore membrane; the electric field strength at the nanopore entrance increased rapidly, exceeding the critical field strength for electroporation (3 kV/cm) up to 33.26 kV/cm at the applied voltage difference of 40 V (Figure 3c).^20^, ^41^, ^42^ Under similar electric conditions, the electric field strength of the bulk EP was 0.15 kV/cm, which is markedly lower than the critical value and is expected to lead to insufficient electroporation.

These results indicated that the nanopore membrane can effectively focus the electric field through the nanopores, thereby enhancing the electric field strength to be sufficiently high for electroporation. Assuming that adherent cells are attached at a distance of approximately 100 nm from the surface,^43^, ^44^, ^45^ sufficient electroporation is expected to occur at 30 V or higher. Based on these simulation studies, we set the voltage conditions for the following in vitro experiments in a range of 10–40 V. Similarly, the electric field focusing on the nanopore membrane can be applied in in vivo electroporation of tissues. The electrical conductivity of tissues (0.08–0.43 S/m) is lower than that of phosphate‐buffered saline (PBS) (1.4 S/m) such that the electric field focusing effect is reduced compared to in vitro conditions (Figure S2).^46^ However, the abrupt change in conductivity near the nanopore can generate a higher electric field strength of 61.54 kV/cm under 40 V, supporting that nEP can be effective for in vivo electroporation of tissues.

### 2.3 In vitro parametric study of nEP

To determine the optimal electroporation conditions, we tested the effects of electric pulse conditions on transfection efficiency, cell recovery, and cell viability. In electroporation, the applied voltage and voltage pulse width are important parameters that determine the rate and duration at which extracellular materials are delivered to the cells. As these two parameters increase, transfection efficiency can be improved; however, excessive electric pulse can cause irreversible cell damage. To find optimal electroporation conditions, 16 electric pulse conditions were applied to a model cell line, MDA‐MB‐231, by coupling a range of voltage intensities from 10 to 40 V and a range of electric pulse widths from 2 to 8 ms. We then evaluated the effectiveness of electroporation by defining the transfection efficiency as the percentage of transfected cells over all cells cultured on the membrane, cell recovery as the percentage of remaining cells on the membrane after electroporation, and cell viability as the percentage of viable cells of the remaining cells. Propidium iodide (PI), a cell‐impermeable nucleic acid dye, was used as a model molecule to evaluate the transfection efficiency. Since cells can be detached from the membrane or burst during electroporation, we evaluated their viability as well as their recovery efficiency.

**FIGURE 4:**
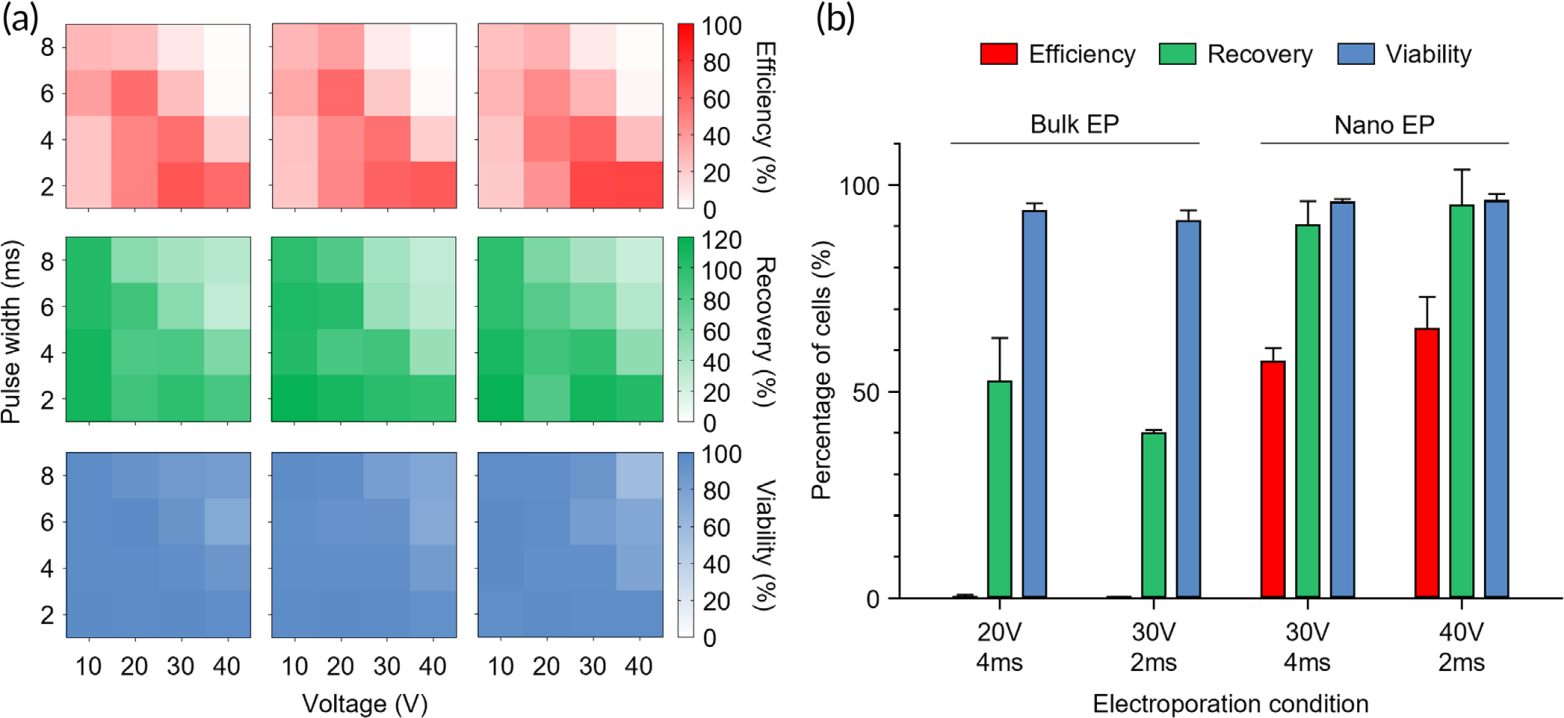
In vitro parametric study to determine optimal electroporation conditions. (a) Effects of 16 electric pulse conditions on transfection efficiency, cell recovery, and cell viability. Each column represents a replicate in the experiment. (b) Comparison between bulk EP and nEP under similar electric pulse conditions. The bulk EP and nEP devices have different inter‐electrode distances of 2 mm and 2.7 mm, respectively. Thus, the electric pulse conditions of 20 and 30 V at the 2 mm inter‐electrode distance correspond to 27 and 40.5 V, respectively (*n* = 3).

As shown in Figure 4a, when the pulse width was as low as 2 ms, the transfection efficiency increased with the voltage intensity, while cell recovery and viability were not considerably affected. However, under a high pulse width of 10 ms, all three efficiency parameters decreased as the voltage increased. These results show that voltage and pulse width are coupled parameters for electroporation, and longer pulse widths above the critical field strength can cause irreversible damage to cells. Based on this parametric study, in the matrix of the electrical pulse conditions, we determined the optimal conditions (30 V, 4 ms, and 40 V, 2 ms) located on the lower right side of the diagonal. After optimizing the electric pulse conditions, we further studied whether nEP was more effective for electroporation than bulk EP (Figure 4b). The results showed that bulk EP at the electric pulse conditions of 20 V, 4 ms, and 30 V, 2 ms were insufficient for transfection (less than 0.8%) due to the electric field strengths (0.10 and 0.15 kV/cm, respectively) being lower than the critical value (3 kV/cm) (Figure 3).^20^, ^41^, ^42^ Nonetheless, the relatively high voltage intensities caused severe electrolysis and cell death in bulk EP, resulting in low cell recoveries of <64.2% (Figure 4b). However, under similar electric pulse conditions, the addition of the nanopore membrane increased the resistance of the entire system while lowering the electric current to prevent electrolysis and cell death. In addition, the localized potential drop across the nanopore membrane dramatically increased the electric field strength above the critical value, thereby ensuring effective electroporation (more than 55.3%) while retaining cell recovery (more than 84.9%) and viability (more than 94.6%).

### 2.4 Characterization of nanopore needle electrode for in vivo nEP

**FIGURE 5:**
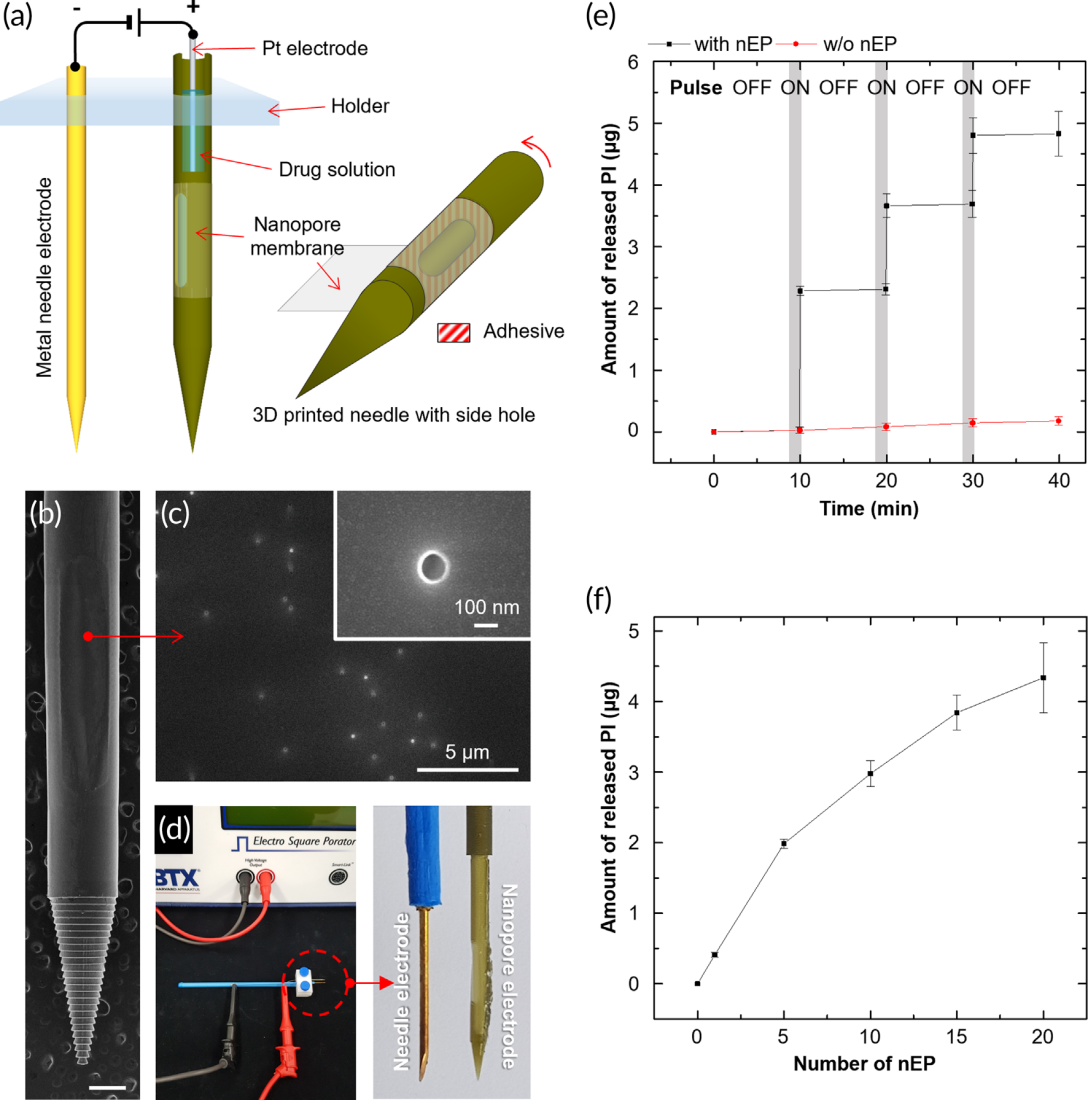
In vivo nEP system with 2‐needle array electrodes. (a) Schematic showing the 2‐needle array electrodes that can be connected to a power supply. The array consists of a conventional metal (Au‐coated stainless steel) needle electrode and a 3D‐printed hollow needle with a side hole (nanopore electrode). The nanopore membrane was bonded using a photocurable adhesive to seal the side hole of the 3D‐printed needle. (b, c) SEM images of (b) the nanopore electrode (scale bar: 500 μm) and (c) nanopore membrane adhered on the open side hole. (d) Photographs of the 2‐needle array electrodes connected to a commercial electroporator. (e, f) In vitro model agent release using the nEP system with 2‐needle array electrodes. (e) Electric pulse‐driven release of a model agent (PI). (f) Amount of released PI from the nanopore electrode depending on the number of nEP (40 V, 2 ms, 99 pulses). (*n* = 3)

To extend the nEP principle identified in the in vitro testing device for in vivo intracellular delivery in deep tissues, we designed 2‐needle array electrodes consisting of a conventional metal (Au‐coated stainless steel) needle electrode and a 3D‐printed hollow needle with a side hole (to be covered with a nanoporous membrane), as illustrated in Figure 5a. The nanoporous membrane was bonded using an adhesive to seal the side hole of the nonconductive 3D‐printed needle. After filling the inner space of the nanopore needle with a solution containing delivery molecules, a Pt wire electrode was inserted into the nanopore needle while it was submerged in the solution. By applying electric pulses through the 2‐needle array electrode, cells close to the nanoporous membrane could be changed to a selectively permeable state, and a target model agent (PI) in the solution could be delivered into the cells through the nanopores. Figure 5b shows the SEM image of a 3D‐printed needle after bonding the nanoporous membrane to the side hole. The needle was made of polyacrylate‐based photocurable resin using digital light processing (DLP)‐based precision 3D printing. It had a conical tip with a diameter of less than 100 μm. The side hole of the needle was tightly sealed by adhering it to the nanopore membrane with a photocurable adhesive without blocking the nanopores (Figure 5c). After fixing the nanopore electrode to face the membrane side toward the metal electrode, the 2‐needle array electrode was connected to a commercial electroporator using a micrograbber cable. The distance between the two electrodes (the metal needle electrode and Pt electrode inside the nanopore needle) was fixed at 2.7 mm (Figure 5d). To confirm the electric pulse‐driven release of PI through the nanopores, we performed in vitro release tests by controlling the nEP conditions. After filling 1 μL of PI solution (PI concentration: 10 mM) into the nanopore electrode, the 2‐needle array electrode connected to the electroporator was submerged in the PBS solution. The release behavior of PI was assessed by measuring the absorbance at 535 nm using a UV/Vis spectrophotometer. Electric pulses of 40 V with a pulse duration of 20 ms were applied 99 times, followed by an interval of 10 min without electrical pulses; this cycle was repeated five times. The interval time between electric pulses was established to confirm electric pulse‐responsive release of PI. Notably, the release profile of PI from the nanopore electrode exactly matched with the actuation of the electrical pulses, highlighting the on‐demand release of target molecules triggered by the low‐voltage electrical stimulus (Figure 5e). In addition, the amount of released PI increased depending on the number of nEP (40 V) (Figure 5f). Due to the limited loading volume of the PI solution in the nanopore needle, the release amount per electrical pulse decreased with an increase in the number of nEP. This electric‐responsive release system would be advantageous for localized treatment to reduce off‐target effects and personalized therapy that requires precise drug release according to the patient's condition.^47^, ^48^, ^49^ In contrast, with the bulk EP system, it is difficult to achieve local, quantitative, and stimuli‐responsive release because electrical stimulation must be applied after the drug solution is injected into the tissue.

### 2.5 Efficacy and safety of in vivo nEP system for intracellular delivery in a pilot study

**FIGURE 6:**
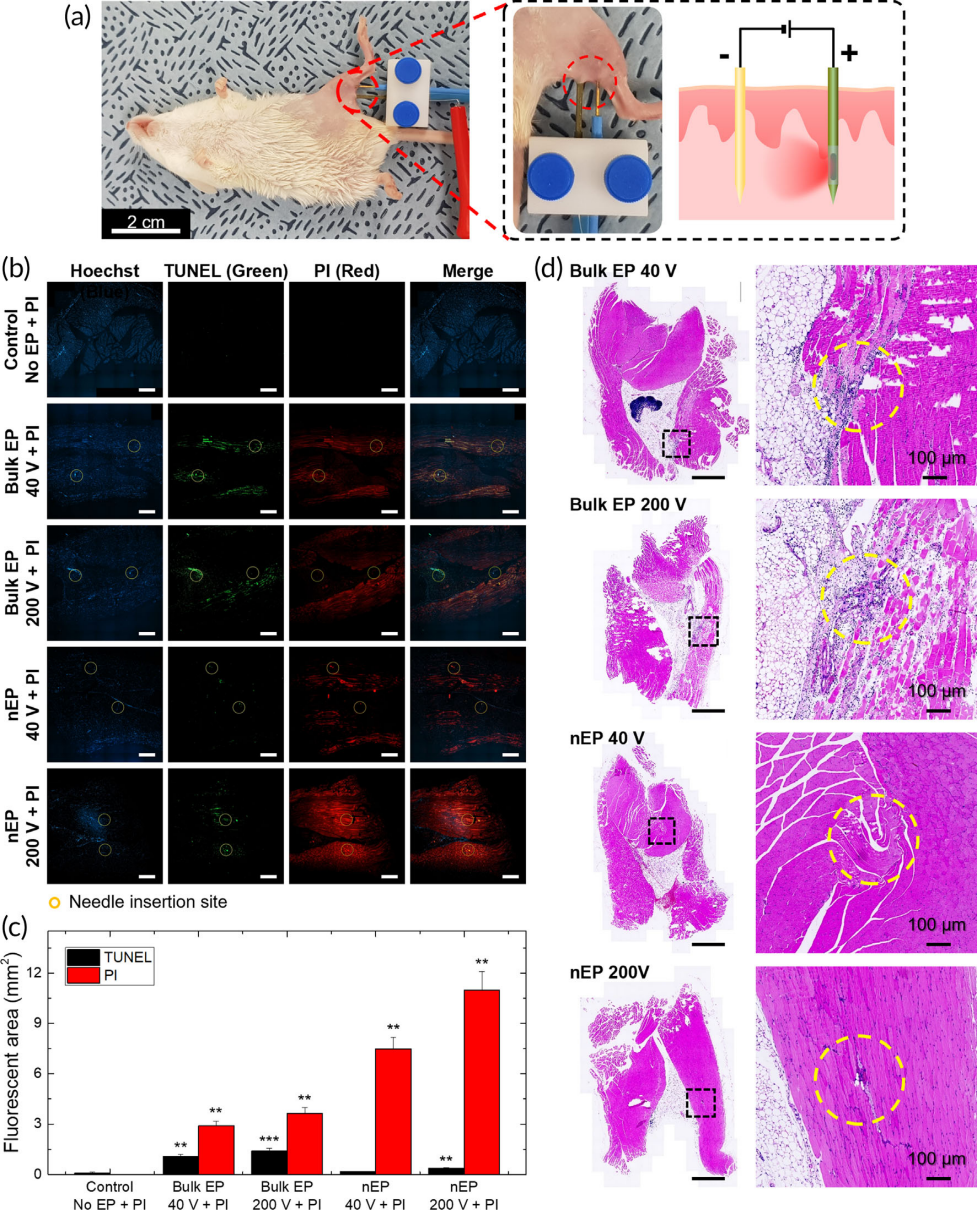
In vivo nEP tests with experimental animals. (a) Photographs showing the in vivo nEP system applied to the thigh muscle of a mouse and schematic illustration showing the nEP‐based intracellular delivery of PI (model agent). (b) Fluorescence micrographs from the sectioned tissue showing tissue damage (TUNEL, green) and transfection efficiency of PI (red). Tissue nuclei were stained with Hoechst (blue). Commercially available two metal needle electrodes were used for bulk EP. The yellow dotted circles indicate the needle insertion sites. (scale bar: 100 μm). (c) Bar graph showing the fluorescent area obtained in (b), representing damaged (TUNEL assay) and transfection (PI) regions. The data are represented as mean ± SD. **p < 0.01, ***p < 0.001 vs. control. (d) Histological images of muscle tissues subjected to electroporation (bulk or nEP) after H&E staining (scale bar: 1 mm).

To confirm the transfection efficiency and safety of the nEP system based on a nanopore electrode, in vivo EP tests were performed following the insertion of the two‐needle array electrode into the thigh muscle of a mouse (Figure 6a). Since 200 V is normally recommended for in vivo bulk EP.^50^, ^51^ two electric pulse conditions (40 V, which was optimized in the in vitro test, and 200 V) were used to evaluate the efficiency of the in vivo nEP system compared with conventional bulk EP. For in vivo bulk EP, the same electric pulse conditions were applied with conventional two‐needle electrodes (10 mm Straight Gold TIP®) after injection of PI between the two electrodes.^52^, ^53^

Transfection efficiency was determined by measuring the fluorescence intensity and area of PI (red) from the tissue sections of EP sites imaged 24 h after EP using confocal microscopy, excluding areas overlapping with cell‐damaged regions (green) (Figure 6b). The damage caused by electrical stimulation of the mouse thigh muscle was confirmed by the terminal deoxynucleotidyl transferase dUTP nick end labeling (TUNEL) assay (green). Nuclei in the tissue sections were stained with Hoechst (blue). In the case of bulk EP, a high level of cell damage occurred in a large area around the needle insertion site at both the low voltage (40 V, 1.08 mm^2^) and high voltage (200 V, 1.41 mm^2^). In contrast, only mild tissue damage was observed at both the low voltage (40 V, 0.17 mm^2^) and high voltage (200 V, 0.37 mm^2^) in the nEP group. Based on the fluorescence area of PI (red), the nEP platform showed better transfection efficiency (40 V, 7.49 mm^2^/200 V, 10.97 mm^2^) compared to bulk EP (40 V, 2.90 mm^2^/200 V, 3.63 mm^2^). As shown in Figure 6c, bulk EP showed at least 4 times higher tissue damage than nEP regardless of voltage, whereas nEP yielded approximately 2.5 times higher transfection efficiency than bulk EP.

To examine whether the nEP system based on the nanopore needle electrode was safe to be used in live animals, we conducted histopathological assessments at the needle insertion site following hematoxylin and eosin (H&E) staining (Figure 6d). Inflammatory cell infiltration and fibrosis were observed in the 40 V bulk EP, and more severe tissue damage by electrical stimulation occurred at the needle insertion site in the 200 V bulk EP. Remarkably, the nEP system showed negligible tissue damage, even under high‐voltage condition (200 V), highlighting the low tissue damage observed during the in vivo electroporation carried out using nEP system in this study. These results demonstrate that the nEP system provides an effective and safe electroporation method for application in deep tissues.

## 3 CONCLUSIONS

We have successfully designed and demonstrated a new nEP system that allows precise on‐demand intracellular delivery of cell‐impermeable molecules in deep tissues with high cell viability and insignificant tissue damage. Simulation studies were performed to screen for optimal electroporation conditions. In vitro studies demonstrated the effectiveness of localized electroporation near the nanopore membrane in terms of high transfection efficiency, cell recovery, and cell viability. Based on the results of in vitro experiments and simulations, a new nanopore needle electrode for an in vivo nEP system was fabricated by combining micro‐ and nano‐manufacturing technologies. In experimental animals, we demonstrated enhanced transfection efficiency and safety of the nEP system, while also showing on‐demand release control of a model agent (PI), which was easily controlled by varying the electric pulse and voltage of nEP. Furthermore, the nEP platform exhibited negligible tissue damage, even at high voltages (>200 V). Thus, this in vivo nEP platform based on the nanopore needle electrode suggests a safe and biocompatible approach for the intracellular delivery of therapeutic agents in deep tissues, which is required for cell reprogramming and treatment of various diseases.

## 4 MATERIAL AND METHODS

### 4.1 Materials

The MDA‐MB‐231 cell line was obtained from the Korean Cell Line Bank (Seoul, Korea). Six‐weeks‐old BALB/c mice were purchased from SAMTACO Bio, Inc. (Osan, Korea). The track‐etched polycarbonate membranes with uniform nanopores of 100 nm and pore density of 10^7^ pores/cm^2^ were purchased from Sterlitech (AL). A 6‐well transwell insert was purchased from SPL Life Sciences (Pocheon, Korea). Roswell Park Memorial Institute (RPMI) 1640 medium, phosphate‐buffered saline (PBS), fetal bovine serum (FBS), and penicillin–streptomycin were purchased from Welgene (Gyeongsan, Korea). Trypsin–EDTA and fibronectin were purchased from Gibco (NY). Propidium iodide (PI) and proteinase K were purchased from Sigma–Aldrich (MO). Hematoxylin and eosin (H&E) were purchased from BioGnost (Zagreb, Croatia). An apoptosis detection kit (mk500) was purchased from Takara (Kyoto, Japan). Hoechst stain was purchased from Invitrogen (MA).

### 4.2 Fabrication of the in vitro nEP device

The in vitro nEP device was fabricated by plating platinum on titanium plates and adhering the platinum electrode plates to the 3D‐printed multi‐well device and chamber lid (Figure 2a). The upper and lower electrodes were arranged such that they had a constant distance of 2.7 mm when the lid was closed. The track‐etched nanopore membrane was adhered to a 6‐well transwell insert with a thermosetting adhesive, PDMS. The surface of the nanopore membrane was then treated with oxygen plasma for 70 s and incubated with a fibronectin solution for 4 h to improve cell adhesion. Cell adhesion experiments showed that cells could not adhere properly without a fibronectin coating due to the hydrophobic nature of the nanopore membrane. When fibronectin was coated, optimal cell adhesion was observed at a concentration of 20 μg/mL or higher. Electroporation experiments were performed after culturing the cells overnight on the nanopore membrane.

### 4.3 Cell culture for in vitro nEP

The MDA‐MB‐231 cell line was cultured in RPMI 1640 medium supplemented with 10% (v/v) FBS and 1% (v/v) penicillin–streptomycin at 37°C in a 5% CO_2_ incubator. MDA‐MB‐231 cells were passaged every 2–3 days using 0.05% trypsin–EDTA. The nanopore membrane in the hanging insert was coated with 20 μg/mL fibronectin at 37°C for 4 h, unless specified. Subsequently, the membrane was washed with PBS, and 3 × 10^5^ cells were plated on the membrane and incubated for 24 h to allow adherence. PI, a cell‐impermeable nucleic acid intercalating dye, was used as a model molecule to evaluate transfection efficiency. For electroporation, 10 μg/mL PI was loaded into the bottom well of the in vitro nEP device, and the hanging insert was then placed into the well. After the Pt‐electrode lid was placed on top of the hanging insert filled with PBS, electric pulses were applied to the device using an electroporation system (ECM 830, BTX). After electroporation, the hanging insert was transferred to a 6‐well plate to stabilize the electroporated cells for 30–60 min in an incubator (37°C). Cell viability was examined by a trypan blue exclusion assay after transfection efficiency analysis.

### 4.4 Flow cytometry analysis of transfected cells

For transfection efficiency analysis, transfected cells were detached using 0.05% trypsin–EDTA, washed twice with PBS, and then resuspended in PBS supplemented with 2% FBS. The cells were analyzed using BD Accuri™ C6 flow cytometer (BD Biosciences). To identify the transfected cells, a nontransfected negative control was used to set the gate line (Figure S3). The fluorescence signals from the transfected cells were detected in the FL2 channel.

### 4.5 Numerical simulation of bulk EP and nEP

Electric field simulations were performed using the finite‐element COMSOL Multiphysics software (COMSOL, Inc.) to visualize the electric field distributions in the bulk EP and nEP devices and calculate the resulting electric field strengths. The 3D finite‐element models for the devices were created in the same dimensions and were then solved using the AD/DC module of COMSOL Multiphysics. The electrical conductivity and relative permittivity were set to 1.4 S/m and 80, respectively, for PBS, and 0.3 S/m and 60,000, respectively, for the tissue.^46^

### 4.6 Fabrication of the nanopore electrode

The nanopore electrode was fabricated by wrapping a nanopore membrane on a side hole needle 3D printed using a high‐resolution DLP 3D printer (Perfactory® Micro Plus HD, EnvisionTEC, Germany) (Figure 5b). The side‐hole needle model was designed using Sketch up (Trimble), and the optimized STL model was sliced into 25 μm using Perfactory Rapid Prototyping (Envision TEC, Germany). A photocurable resin (HTM‐140, EnvisionTEC, Germany) was used for 3D printing. To wrap the side of the 3D‐printed needle with a nanopore membrane, a UV‐curable adhesive was thinly applied on the 3D‐printed needle, and then the nanopore membrane was wrapped on the needle and UV‐irradiated. The nanopore membrane was adhered to a 3 × 0.5 mm side hole of the 3D‐printed needle. The surface of the membrane wrapping the nanopore electrode was observed by field‐emission scanning electron microscopy (FE‐SEM; S‐4700, Hitachi, Japan); pore blockage by the adhesive was not observed. The interior of the nanopore electrode was in the form of a column with a diameter of 500 μm, which could be filled with PI solutions. To apply a pulse to the nanopores, a Pt wire electrode was inserted into the interior of the nanopore electrode.

### 4.7 Evaluation of the release of model agent from nanopore electrode in in vitro test

The amount of PI released from the nanopore electrode was measured to evaluate the release of the model agent through the electrode. After connecting the nanopore electrode filled with 10 mM PI solution to the electroporator (ECM 830, BTX), it was immersed in 200 μL of PBS along with the counter electrode. Electric pulses were applied to the nanopore electrode, and the concentration of PI released in PBS was measured using a UV/Vis spectrophotometer (Libra S70, Biochrom, UK).

### 4.8 Animals

The animal protocol used in this study was reviewed and approved based on ethical procedures and scientific care by the Pusan National University‐Institutional Animal Care and Use Committee (approval code: PNU‐2019‐2347). Six‐weeks‐old BALB/c mice purchased from SAMTACO Bio Inc. (Osan, Korea) and acclimated for a week. During the experiment, all mice were housed in standard cages and free access to sterilized water and pellets. All animals were maintained at a temperature of 22 ± 2°C with humidity of 55 ± 5% and a light/dark cycle of 12 h/12 h.

### 4.9 In vivo nEP test using nanopore electrode

To evaluate the transfection efficiency and safety of the nanopore electrode in in vivo experiments, each electrode was inserted into the shaved hindlimb thigh muscle of the mouse, and an electric pulse was applied. PI was used as the model agent. In the case of bulk EP, 10 μL of 1 mM PI was injected intramuscularly into the EP site before applying the pulses, and in the case of nEP, 1 μL of 10 mM PI was injected into the nanopore electrode. Both the pulse conditions commonly used for bulk EP (200 V, 20 ms, 4 times) and optimized for nEP (40 V, 2 ms, 99 times) were applied to each electrode. During electrical stimulation, the experimental animals were anesthetized using an anesthesia breathing system.

### 4.10 Histopathological analysis

To evaluate the histopathological changes, the thigh muscle of each mouse was freshly excised, fixed in 10% neutral buffered formalin for 24 h, and embedded in paraffin. Paraffin‐embedded specimens were sliced perpendicular to the electrode insertion direction into 5‐μm‐thick sections. Deparaffinized skin sections were stained with H&E for analyzing tissue damage. Bright‐stained sections were then examined with a digital fluorescence slide scanner (Axio Scan Z1, ZEISS, Germany) to assess histological changes, including damaged tissue.

### 4.11 TUNEL assay

Apoptosis was evaluated using an in situ apoptosis detection kit (mk500, Takara, Japan). Proteolysis was performed using proteinase K for 15 min. Working Strength TdT enzyme was added to the proteolyzed slices (5 μL TdT enzyme: 45 μL reaction buffer). Then, the slices were incubated in a humidified chamber at 37°C for 90 min. Next, the slices were treated with fluorescein isothiocyanate‐antibody (FITC‐Ab) mixture in a humidified chamber at 37°C for 60 min. Finally, sections counterstained with Hoechst were observed using a digital fluorescence slide scanner. The areas with fluorescence were determined using the ImageJ 1.50e software (Bethesda).

## AUTHOR CONTRIBUTIONS

**Gyeong Won Lee:** Conceptualization (equal); data curation (equal); investigation (equal); methodology (equal); writing – original draft (equal); writing – review and editing (equal). **Byeongyeon Kim:** Data curation (equal); investigation (equal); methodology (equal); writing – original draft (equal). **Tae Wook Lee:** Investigation (equal); methodology (equal). **Sang‐Gu Yim:** Conceptualization (supporting); data curation (equal); investigation (equal). **Ajeesh Chandrasekharan:** Data curation (equal); investigation (equal). **Hyewon Kim:** Data curation (equal); methodology (equal). **Sungyoung Choi:** Conceptualization (equal); funding acquisition (equal); supervision (equal); writing – original draft (equal); writing – review and editing (equal). **Seung Yun Yang:** Conceptualization (equal); funding acquisition (equal); supervision (equal); writing – original draft (equal); writing – review and editing (equal).

## CONFLICT OF INTEREST

The authors declare that they have no conflict of interest.

### PEER REVIEW

The peer review history for this article is available at https://publons.com/publon/10.1002/btm2.10418.

## Supporting information


**Figure S1** Pore size distribution in the nanopore membrane.
**Figure S2** Simulation results for in vivo electroporation. (a) There is no significant difference in the electric field distribution and strength between in vivo and in vitro settings for bulk electroporation due to the homogeneous medium composition. (b) A significant voltage drop occurs along the tissue in the in vivo nEP due to its low conductivity, compared to that of PBS. (c, d) The applied voltage was 40 V for both bulk EP and nEP. Electric field strength profiles along the direction away from (c) a certain point in the bulk EP and (d) the nanopore entrance in the nEP.
**Figure S3** Gating strategy to identify transfected cells.

## Data Availability

The data that support the findings of this study are available from the corresponding authors upon reasonable request.
