# Supplementary material for: Nanoporous electroporation needle for localized intracellular delivery in deep tissues
Source: Bioeng Transl Med. 2022 Dec 8;8(4):e10418. doi: 10.1002/btm2.10418 (PMC10354752; doi:10.1002/btm2.10418)
Supplement: Supplementary file 1 — Figure S1 Pore size distribution in the nanopore membrane. Figure S2 Simulation results for in vivo electroporation. (a) There is no significant difference in the electric field distribution and strength between in vivo and in vitro settings for bulk electroporation due to the homogeneous medium composition. (b) A significant voltage drop occurs along the tissue in the in vivo nEP due to its low conductivity, compared to that of PBS. (c, d) The applied voltage was 40 V for both bulk EP and nEP. Electric field strength profiles along the direction away from (c) a certain point in the bulk EP and (d) the nanopore entrance in the nEP. Figure S3 Gating strategy to identify transfected cells. [file BTM2-8-e10418-s001.docx]

Supporting information

Nanoporous electroporation needle for localized intracellular delivery in deep tissues

Gyeong Won Lee^1^, Byeongyeon Kim^2^, Tae Wook Lee^1^, Sang-Gu Yim^1^, Ajeesh Chandrasekharan^1^, Hyewon Kim^2^, Sungyoung Choi^2,*^, Seung Yun Yang^1,*^

^1^Department of Biomaterials Science (BK21 Four Program), Pusan National University, Miryang 50463, Korea

^2^Department of Biomedical Engineering, Department of Electronic Engineering, Hanyang Institute of Bioscience and Biotechnology, Hanyang University, Seoul 04763, Korea

Correspondence: Seung Yun Yang ([syang@pusan.ac.kr](mailto:syang@pusan.ac.kr)) or Sungyoung Choi ([sungyoung@hanyang.ac.kr](mailto:sungyoung@hanyang.ac.kr))

KEYWORDS: Electroporation, nanopore membrane, intracellular delivery, electric pulse-driven drug delivery


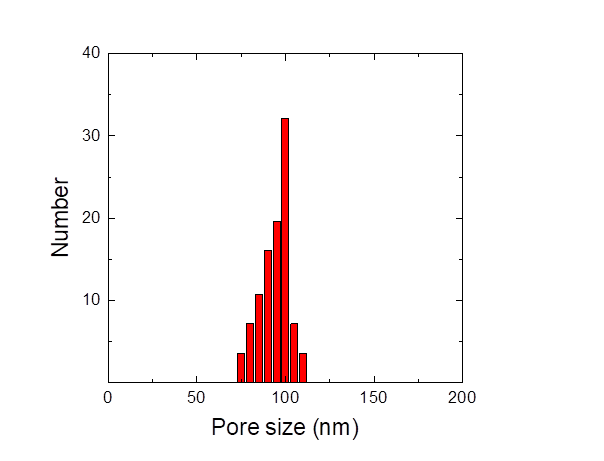


**Fig S1**. Pore size distribution in the nanopore membrane.


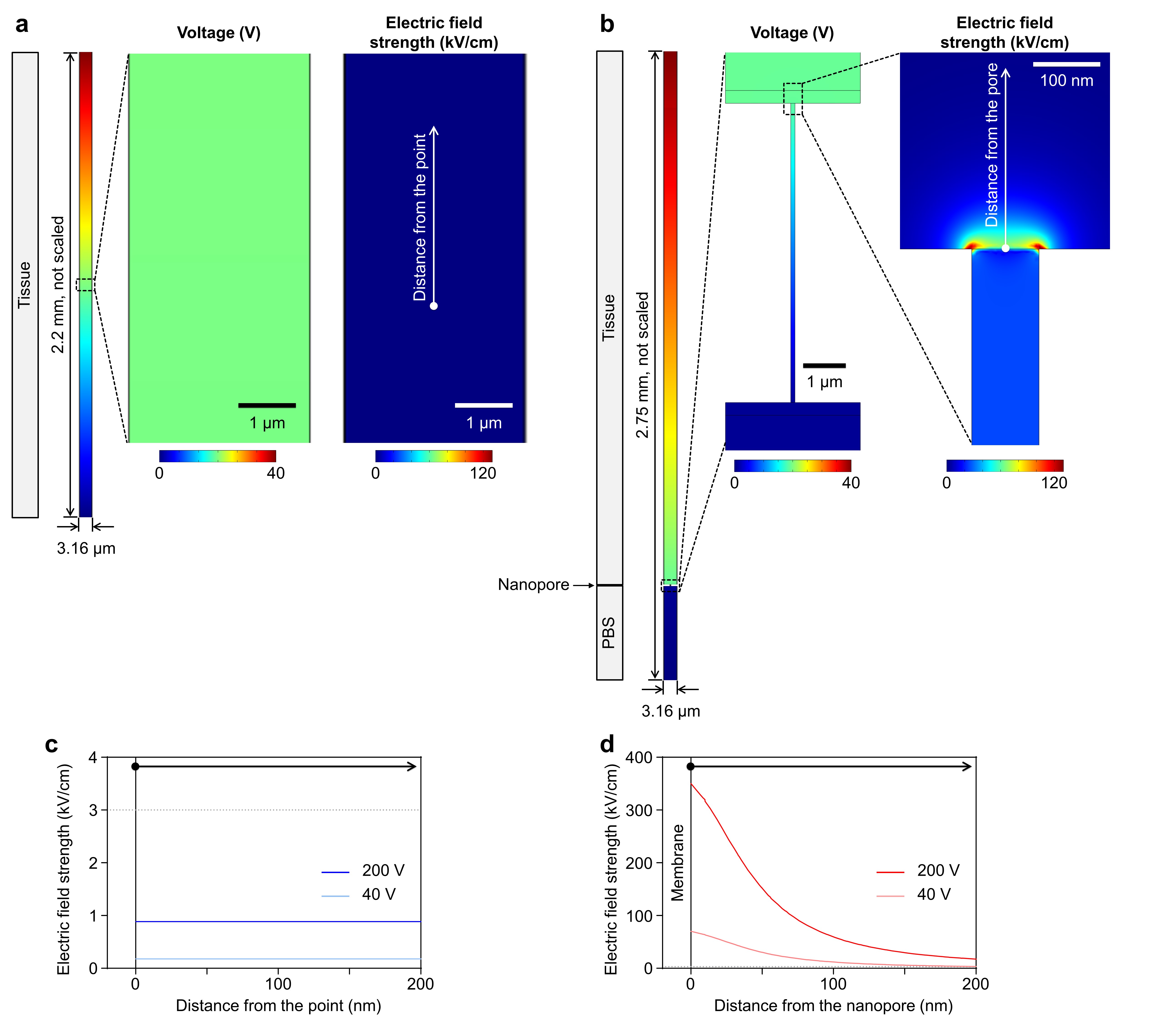


**Fig. S2**. Simulation results for *in vivo* electroporation. (a) There is no significant difference in the electric field distribution and strength between *in vivo* and *in vitro* settings for bulk electroporation due to the homogeneous medium composition. (b) A significant voltage drop occurs along the tissue in the *in vivo* nEP due to its low conductivity, compared to that of PBS. (c and d) The applied voltage was 40 V for both bulk EP and nEP. Electric field strength profiles along the direction away from (c) a certain point in the bulk EP and (d) the nanopore entrance in the nEP.


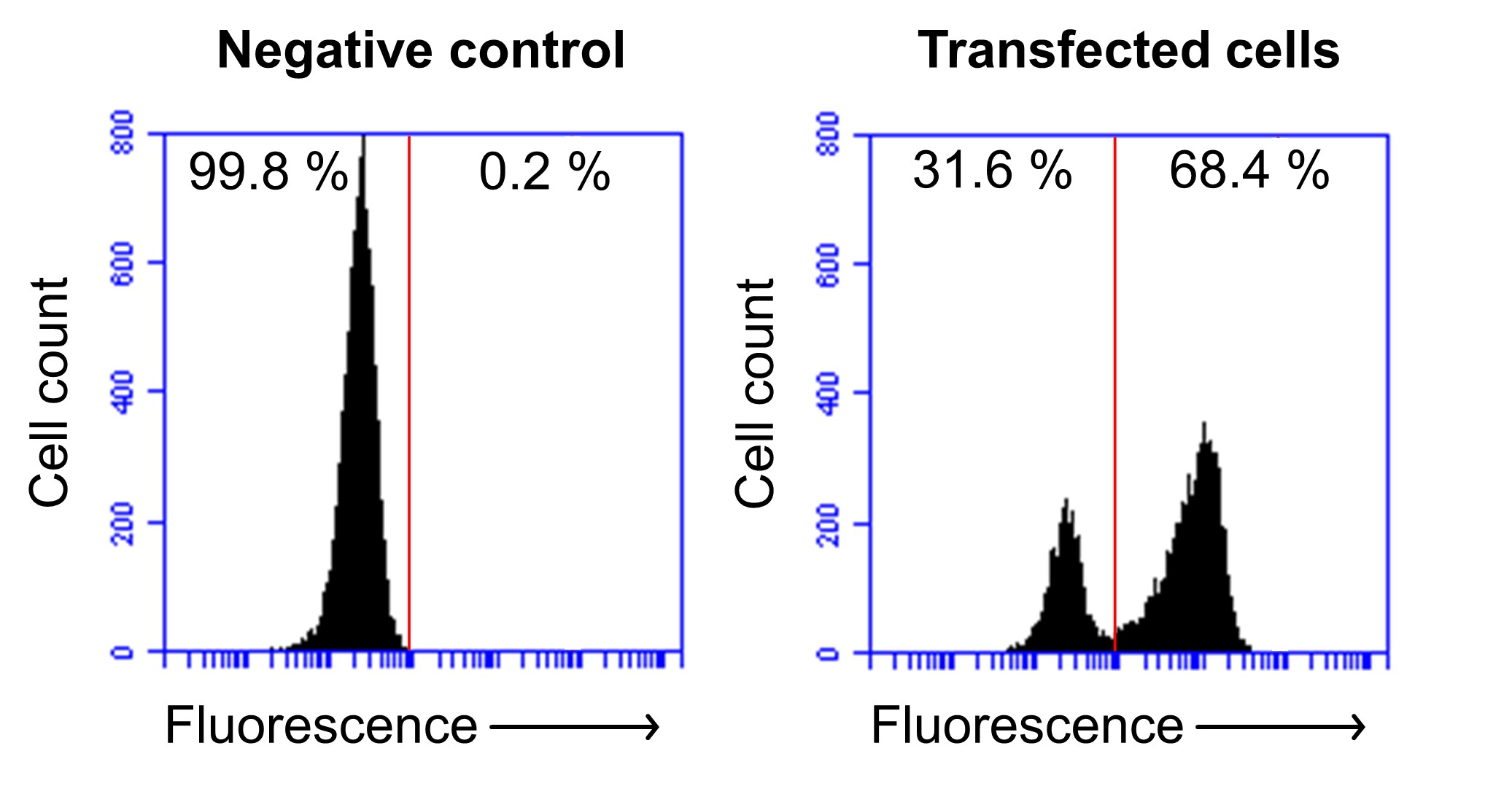


**Figure S3**. Gating strategy to identify transfected cells.
